# Human sapovirus GI.2 and GI.3 from children with acute gastroenteritis in northern Brazil

**DOI:** 10.1590/0074-02760180574

**Published:** 2019-04-04

**Authors:** Audrey Cilli, Adriana Luchs, Elcio Leal, Danielle Gill, Flavio Augusto de Pádua Milagres, Shirley Vasconcelos Komninakis, Rafael Brustulin, Maria da Aparecida Rodrigues Teles, Márcia Cristina Alves Brito Sayão Lobato, Rogério Togisaki das Chagas, Maria de Fátima Neves dos Santos Abrão, Cassia Vitória de Deus Alves Soares, Xutao Deng, Eric Delwart, Ester Cerdeira Sabino, Antonio Charlys da Costa

**Affiliations:** 1 Instituto Adolfo Lutz, Centro de Virologia, Núcleo de Doenças Entéricas, São Paulo, SP, Brasil; 2 Universidade Federal do Pará, Instituto de Ciências Biológicas, Belém, PA, Brasil; 3 Universidade de São Paulo, Instituto de Medicina Tropical, São Paulo, SP, Brasil; 4 Universidade de São Paulo, Faculdade de Medicina, LIM/46, São Paulo, SP, Brasil; 5 Universidade Federal de Tocantins, Palmas, TO, Brasil; 6 Laboratório Central de Saúde Pública do Tocantins, Palmas, TO, Brasil; 7 Secretaria da Saúde do Tocantins, Palmas, TO, Brasil; 8 Faculdade de Medicina do ABC, Programa de Pós-Graduação em Ciências da Saúde, Santo André, SP, Brasil; 9 Universidade Federal de São Paulo, Laboratório de Retrovirologia, São Paulo, SP, Brasil; 10 Blood Systems Research Institute, San Francisco, CA, USA; 11 University of California, Department Laboratory Medicine, San Francisco, CA, USA

**Keywords:** Caliciviridae, sapovirus, gastroenteritis, genotypes, deep sequencing

## Abstract

Human sapoviruses (HSaV) are considered important causative agents of acute gastroenteritis in humans worldwide. However, knowledge of the genetic characteristics of the whole genome of HSaV in Brazil is limited. Here we report the complete genome sequences of six HSaVs GI.2 and two GI.3 strains obtained from children with acute gastroenteritis in the Northern region of Brazil. Next generation sequencing was used to obtain the full genome and molecular characterization of the genome was performed. Phylogenetic analysis of the genome was also performed. Only one complete HSaV GI.2 genome characterization in the country precedes that of the present study. This is the first complete genome sequence of genotype GI.3 in Brazil. The data obtained in this investigation can contribute to the augmentation of the database on the molecular diversity of HSaVs strains circulating in Brazil, and to the improvement of current typing protocols.

Human sapoviruses (HSaVs) belong to the *Sapovirus* genus of the family *Caliciviridae* are considered important causative agents of acute gastroenteritis (AGE) in humans.^1^ ^,^ ^2^ Mortality associated with this pathogen is rare and the symptoms are generally mild.^3^ Based on the VP1 nucleotide sequence, human HSaVs are classified into genogroups GI, GII, GIV, and GV, and subdivided into 17 genotypes,^1^ with an additional proposed GII.8 genotype.^4^ HSaV has a positive-sense, single stranded RNA genome of 7.1-7.7kb in length which contains two open reading frames (ORFs). ORF1 encodes a large polyprotein containing the nonstructural proteins followed by the major capsid protein VP1. ORF2 is predicted to encode the minor structural protein VP2.^1^

Although HSaV has been accepted as one of the causes of acute gastroenteritis worldwide, little is known about the genetic characteristics of HSaV in Brazil based on whole genome analysis. In Brazil, the most common genotypes identified are GI.1, GI.2 and GII.1.^5^ ^,^ ^6^ ^,^ ^7^ ^,^ ^8^ Here we report the complete genome sequences of six HSaVs GI.2 and two GI.3 strains obtained from children with acute gastroenteritis in the Northern region of Brazil. Phylogenetic analysis was performed for comparison with other previously reported genogroups/genotypes.

The samples BRA/TO-07, BRA/TO-31, BRA/TO-48, BRA/TO-49, BRA/TO-65, BRA/TO-66, BRA/TO-89 and BRA/TO-90 were obtained from the Central Laboratory of Public Health of Tocantins state (LACEN/TO), located in the Northern region of Brazil (Table). All patients were experiencing acute gastroenteritis symptoms, such as diarrhea, vomiting and fever.

The protocol used to perform deep sequencing was a combination of several protocols normally applied to viral metagenomics and/or virus discovery,^9^ and has been partially described by da Costa et al.^10^ In summary, 50 mg of each human fecal sample was diluted in 500 μL of Hanks’ buffered salt solution (HBSS), added to a 2 mL impact-resistant tube containing lysing matrix C (MP Biomedicals, USA), and homogenized in a FastPrep-24 5G Homogenizer (MP biomedicals, USA). The homogenized sample was centrifuged at 12,000×*g* for 10 min, and approximately 300 μL of the supernatant was then percolated through a 0.45 μm filter (Merck Millipore, Billerica, MA, USA) in order to remove eukaryotic and bacterial cell-sized particles. Approximately, 100 μL, roughly equivalent to one-fourth of the volume of the tube of cold PEG-it Virus Precipitation Solution (System Biosciences, CA, USA) was added to the obtained filtrate, and the contents of the tube were gently mixed then incubated at 4ºC for 24 h. After the incubation period, the mixture was centrifuged at 10,000×*g* for 30 min at 4ºC. Following centrifugation, the supernatant (~350 μL) was discarded. The pellet rich in viral particles was treated with a mixture of nuclease enzymes (14 uni TURBO Dnase and 7 uni RNase Cocktail Enzyme Mix-Thermo Fischer Scientific, CA, USA; 9 uni Baseline-ZERO DNase - Epicentre, WI, USA; 25 Benzonase - Darmstadt, Germany; and 9 RQ1 RNase- Free DNase and 0.09mg RNase A Solution - Promega, WI, USA) in order to digest unprotected nucleic acids. The resulting mixture was subsequently incubated at 37ºC for 2 h.

After incubation, viral nucleic acids were extracted using ZR & ZR-96 Viral DNA/RNA Kit (Zymo Research, CA, USA) according to the manufacturer’s protocol. The cDNA synthesis was performed with AMV Reverse transcription (Promega, WI, USA). A second strand of cDNA was synthesized using DNA Polymerase I Lar e (Klenow) Fragment (Promega, WI, USA). Subsequently, a Nextera XT Sample Preparation Kit (Illumina, CA, USA) was used to construct a DNA library, identified using dual barcodes. For size range, Pippin Prep (Sage Science, Inc.) was used to select a 300 bp insert (range 200-400 bp). The library was deep-sequenced using the HiSeq 2500 Sequencer (Illumina, CA, USA) with 126 bp ends. Bioinformatic analysis was performed according to the protocol previously described by Deng et al.^11^ Contigs that shared percent nucleotide identities of 95% or less were assembled from the obtained sequence reads by *de novo* assembly. The contigs included the group A rotavirus sequences and others, such as enteric viruses (i. e., enterovirus, adenovirus, norovirus), and human, fungal, and bacterial sequences. The resulting singlets and contigs were analyzed using BLASTx to search for similarity to viral proteins in GenBank’s Virus RefSeq. The contigs were compared to the GenBank nonredundant nucleotide and protein database (BLASTn and BLASTx).

Total of 110,104; 38,780; 748,755; 395,031; 28,399; 27,971; 25,835; and 21,539 paired-end reads were obtained from the BRA/TO-07, BRA/TO-31, BRA/TO-48, BRA/TO-49, BRA/TO-65, BRA/TO-66, BRA/TO-89 and BRA/TO-90 samples, respectively. Of the total reads, 6.8% (n = 7,472) from BRA/TO-07, 19.1% (n = 7,402) from BRA/TO-31, 1.1% (n = 8,032) from BRA/TO-48, 1.9% (n = 7,667) from BRA/TO-49, 26% (n = 7,337), 26.6% (n = 7,443) from BRA/TO-66, 28.9% (n = 7,471) from BRA/TO-89 and 34.7% (n = 7,476) from BRA/TO-90 showed BLASTx score (coverage 1856x, 660x, 11745x, 6491x, 485x, 473x, 435x and 363x, respectively) to HSaV. The final genome analysis was performed using Geneious software v9.1.8 (Biomatters Ltd., Auckland, New Zealand). Open reading frames were predicted with the Geneious ORF finder. Based on the bioinformatics pipeline used,^11^ no reads related to human, fungal, or bacterial sequences were obtained.

A public accessible typing tool (http://www.rivm.nl/mpf/norovirus/typingtool) was used to assign the genogroup of the study strains.^12^ Sequences generated here and a set of cognate sequences of SaV available in GenBank were aligned using the BioEdit sequence alignment editor (version 7.0.5.2) program. Genetic analysis was performed with MEGA software version 6.0.^13^ The Kimura two-parameter substitution model and neighbour-joining method was selected to infer phylogenetic relationships among relevant strains. Nucleotide sequences determined in this study have been deposited in GenBank under the accession numbers MK250983-MK250990.

The eight HSaVs strains were classified in genogroup GI (ORF 1) based on the web tool analysis. Phylogenetic tree indicated that six HSaVs samples belong to genotype GI.2 (BRA/TO-07, BRA/TO-31, BRA/TO-65, BRA/TO-66, BRA/TO-89 and BRA/TO-90), and two HSaVs samples (BRA/TO-48 and BRA/TO-49) belong to genotype GI.3 (Table, Figure). Brazilian HSaV GI.2 sequences showed 98.4-99.9% similarity at nucleotide level (nt) (97.8-100% aa) between them, and 92.1-94.3% nt (80.0-84.3% aa) when compared to representative GI.2 strains detected in Brazil, United States, China and Ireland. The Brazilian HSaV GI.3 strains were close related to each other, since they shared 97.0% nt identity (94.6% aa). BRA/TO-48 and BRA/TO-49 GI.3 HSaVs strains exhibited high nucleotide and amino acid identity to the human strain OH08021, isolated in Japan in 2008 (90.5-93.3% nt; 86.5-91.3% aa). When compared with human GI.3 strain isolated in Kenya in 2006, strains BRA/TO-48 and BRA/TO-49 displayed lower genetic homology (87.4-88.2% nt; 73.8-75.2% aa).

Several studies demonstrate HSaV GI.2 as one of the main genotypes associated with outbreaks and sporadic cases of AGE,^14^ ^,^ ^15^ ^,^ ^16^ including in Brazil.^6^ ^,^ ^17^ The data obtained here confirmed the epidemiological role of HSaV GI.2 genotype in AGE etiology. HSaV GI.3 genotype appears to be detected more sporadically.^18^ ^,^ ^19^ In Brazil, a recent study conducted in a day-care center in the Midwest region reported the detection of HSaV GI.3 genotype in asymptomatic children.^7^ There is relatively limited sequence information about Brazilian HSaVs strains at the complete genome level. Before the present study, only one complete HSaV GI.2 genome had been characterized in the country. To the best of our knowledge these are the first complete genome sequences of genotype GI.3 in Brazil.

The data acquired in this investigation can contribute to the growing database on the molecular diversity of HSaV circulating in Brazil and also to future epidemiological studies of HSaV by providing data necessary for the development of more sensitive and specific diagnostic tools that could be used to define the worldwide distribution of the virus.

**Figure:**
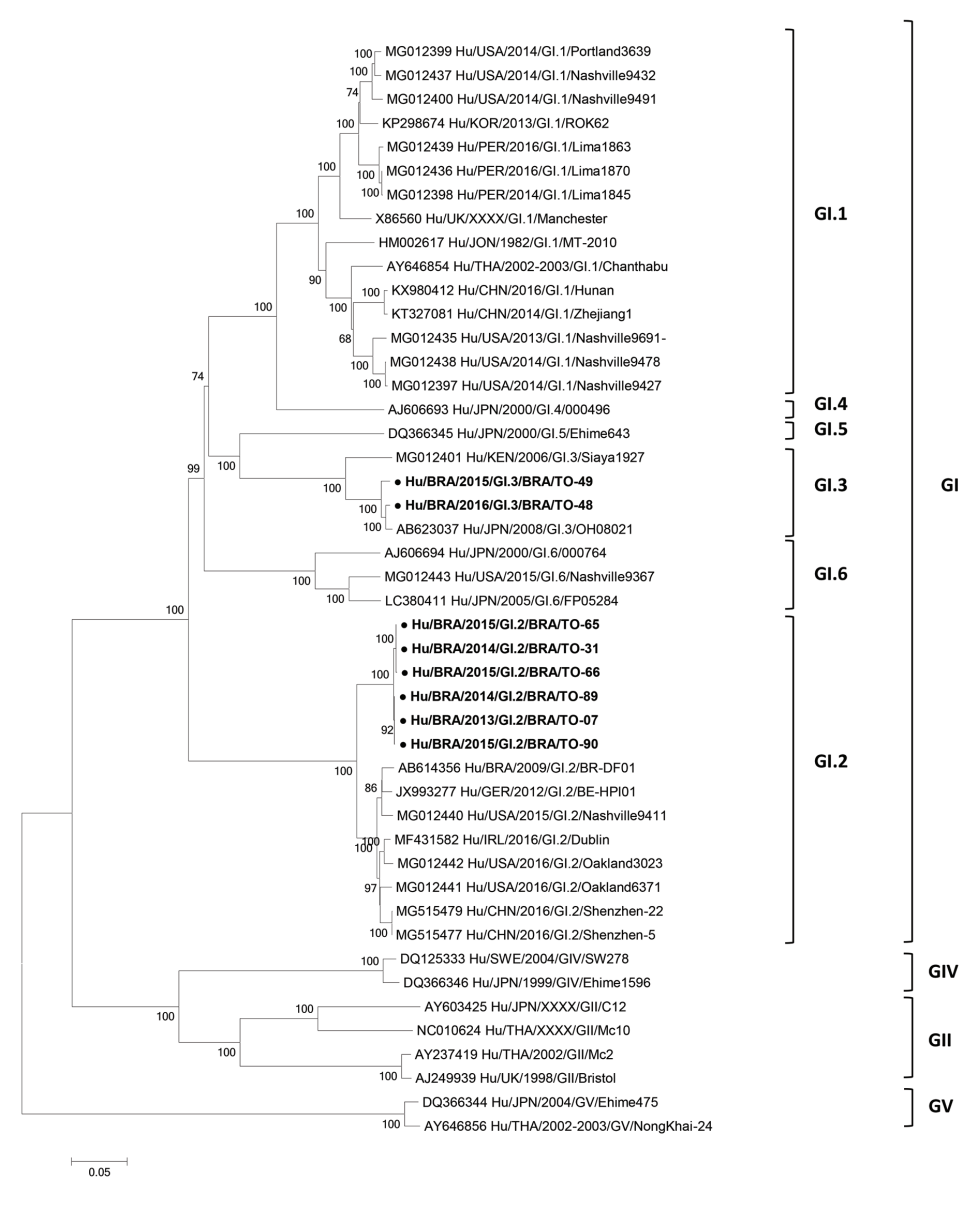
Neighbor-joining phylogenetic tree of nucleotide sequence generate with MEGA 6.0 software of the human sapoviruses (HSaV) strains detected from children with acute gastroenteritis in Brazil (highlighted in bold and ●). References of HSaVs were obtained from GenBank database. Genogroups, genotypes, accession number, isolates, countries and year of each strain are indicated. The scale indicates the number of divergent nucleotide residues. Percentage of bootstrap values is shown at the branch node.

**TABLE:** Socio-demographic data of positive sapoviruses (SaV) samples from patients with acute gastroenteritis in Brazil

Age	Sex	Municipality	State	Month / Year	Genotype	Strain	Accession numbers
7 year	M	Novo Acordo	TO	October/2013	GI.2	BRA/TO-07	MK250986
11 months	F	Fortaleza do Tabaco	TO	May/2014	GI.2	BRA/TO-31	MK250984
1 year	F	Araguaína	TO	January/2016	GI.3	BRA/TO-48	MK250989
10 months	M	Couto Magalhães	TO	September/2015	GI.3	BRA/TO-49	MK250990
9 months	F	Araguaína	TO	August/2015	GI.2	BRA/TO-65	MK250983
9 months	M	Araguaína	TO	February/2015	GI.2	BRA/TO-66	MK250985
4 years	F	Araguaína	TO	April/2014	GI.2	BRA/TO-89	MK250987
5 months	M	Araguaína	TO	July/2015	GI.2	BRA/TO-90	MK250988

TO: state of Tocantins, Brazil.

*Ethics* - Previous Ethics Committee approval was granted by Faculdade de Medicina da Universidade de São Paulo (CAAE: 53153916.7.0000.0065), and Centro Universitário Luterano de Palmas ― ULBRA (CAAE: 53153916.7.3007.5516). This was an anonymous unlinked study, and informed consent was not required according to resolution 466/12 concerning research involving humans (Conselho Nacional de Saúde/Ministério da Saúde, Brasília, 2012).
